# Primary immune regulatory disorders: Undiagnosed needles in the haystack?

**DOI:** 10.1186/s13023-022-02249-1

**Published:** 2022-03-03

**Authors:** Aisling M. Flinn, Andrew R. Gennery

**Affiliations:** Translational and Clinical Research Institute, Faculty of Medical Sciences, Newcastle University, Newcastle upon Tyne, UK

## Abstract

Primary Immune Regulatory Disorders (PIRD) describe a group of conditions characterized by loss of normal inflammatory control and immune tolerance mechanisms, with autoimmunity as a predominant clinical feature. PIRD can arise due to defects in the number or function of regulatory T-lymphocytes, defects in the immune mechanisms required to ‘turn off’ inflammation such as in perforin-dependent cytotoxicity or alterations in cytokine signalling pathways. Diagnosis of PIRD is a significant challenge to physicians due to their rarity, complexity, and diversity in clinical manifestations. Many of these individual conditions lack a genotype–phenotype correlation and display incomplete penetrance. However, establishing a diagnosis is integral in optimizing patient management, including the use of individualized treatment approaches. Increasing awareness among physicians is necessary as patients are likely to present to different subspecialties. Due to the rarity of these conditions, worldwide collaboration and data-sharing is essential to improve our knowledge of the clinical spectrum and disease course in PIRD, and to optimize therapeutic strategies including identification of which patients can benefit from hematopoietic stem cell transplant.

## Introduction

Historically, the term Primary Immune Deficiency (PID) described a genetic disorder causing loss or gain of function in an encoded protein with subsequent immune system dysfunction associated with susceptibility to recurrent, severe or opportunistic infections. Advances in our understanding accompanied by enhanced diagnostic tools over the last decade has made clear that the spectrum of clinical manifestations associated with PID is more extensive, encompassing autoimmunity, allergy, autoinflammation, malignancy and non-malignant lymphoproliferation as well as predisposition to infection leading to the term ‘Inborn Error of Immunity’ (IEI) to better describe this diverse collection of diseases. It is now recognized that certain IEIs exhibit a predominant phenotype of immune dysregulation; this group of disorders has been collectively termed ‘Primary Immune Regulatory Disorders’ (PIRD) which are characterized by loss of normal inflammatory control and self-tolerance mechanisms within the immune system leading to autoinflammation, autoimmunity and lymphoproliferation (Table 1). Patients with these conditions may still develop infections but this is typically a less predominant feature and infections often occur as a complication of end organ damage as a consequence of immune dysregulation.

**Table 1:** Conditions characterized by immune dysregulation

Group	Disorder	Gene	Inheritance	Typical features
Regulatory T-lymphocyte defect (absent/reduced Tregs and/or functional Treg defect)	IPEX syndrome (immune dysregulation, polyendocrinopathy, enteropathy, X-linked)	*FOXP3*	XL	Autoimmune enteropathy, early onset type 1 diabetes mellitus, eczema, thyroiditis, hemolytic anemia, thrombocytopenia, elevated IgE
	CTLA4 haploinsufficiency	*CTLA4*	AD	Autoimmunity particularly cytopenias, enteropathy, type 1 diabetes, lymphoproliferation, interstitial lung disease, recurrent infections, hypogammaglobulinemia
	LRBA deficiency	*LRBA*	AR	Autoimmunity particularly cytopenias, lymphoproliferation, recurrent infections, enteropathy
	STAT3 GOF	*STAT3*	AD	Autoimmunity, lymphoproliferation, infections, short stature
	CD25 deficiency	*IL2RA*	AR	Autoimmunity, lymphoproliferation
Autoimmune Lymphoproliferative Syndromes (ALPS)	ALPS-FAS (most common)	F*AS*	AD or AR	Lymphadenopathy, splenomegaly, hepatomegaly, autoimmune cytopenias, increased risk of lymphoma, defective lymphocyte apoptosis, elevated double negative T-cells. In ALPS-FAS, elevated soluble Fas Ligand, IL-10, vitamin B12, IgG
	ALPS-FASLG	*TNFRSF6*	AR	
	ALPS-Caspase10	*CASP10*	AD	
	ALPS-Caspase8	*CASP8*	AR	
HLH – familial disorders	Perforin deficiency	*PRF1*	AR	Early-onset HLH, fever, hepatosplenomegaly, cytopenias, decreased/absent NK and CTL activity
	Munc13-4 deficiency	*UNC13D*	AR	
	Syntaxin 11 deficiency	*STX11*	AR	
	Munc18-2 deficiency	*STXBP2*	AR	
HLH—IEI syndromes associated with increased incidence of HLH	Chediak Higashi syndrome	*LYST*	AR	Recurrent infections, partial oculocutaneous albinism, progressive neurological dysfunction, neutropenia, giant cytoplasmic granules
	Griscelli syndrome type 2	*RAB27A*	AR	Partial albinism, neutropenia, thrombocytopenia, neurological impairment
	Hermansky Pudlak syndrome type 2	*AP3B1*	AR	Oculocutaneous albinism, bleeding diathesis, neutropenia, hearing loss, pulmonary fibrosis
	Hermansky Pudlak syndrome type 10	*AP3D1*	AR	Oculocutaneous albinism, bleeding diathesis, microcephaly, neurodevelopmental delay
	X-linked lymphoproliferative disease 1	*SH2DIA*	XL	EBV-associated HLH, lymphoma, aplastic anemia
	X-linked lymphoproliferative disease 2	*XIAP*	XL	EBV-associated HLH, colitis, hepatitis
Immune dysregulation with colitis/inflammatory bowel disease	IL-10 deficiency	*IL10*	AR	Early onset severe colitis, respiratory tract infections, folliculitis, arthritis
	IL-10 receptor deficiency	*IL10RA* *IL10RB*	AR	
	TGFB1 deficiency	*TGFB1*	AR	Recurrent viral infections, microcephaly, encephalopathy
	RIPK1 deficiency	*RIPK1*	AR	Recurrent infections, arthritis
	NFAT5 haploinsufficiency	*NFAT5*	AD	Colitis, recurrent respiratory tract infections
Innate	STAT1 GOF	*STAT1*	AD	Chronic mucocutaneous candidiasis, recurrent infections, autoimmunity
	NFKB2 deficiency	*NFKB2*	AD	Recurrent sinopulmonary infections, endocrinopathies, low immunoglobulins
Thymic disorder	APECED	*AIRE*	AD or AR	Adrenal insufficiency, hypoparathyroidism, hypothyroidism, other autoimmunity, chronic mucocutaneous candidiasis

An increasing number of monogenetic disorders with immune dysregulation have been identified. These disorders may arise due to defects in Tregs, central tolerance, control of inflammatory responses and lymphocyte apoptosis, and in cytokine signalling pathways. This table is a non-exhaustive list of some of the more common PIRDs known to date

## Underlying pathophysiology in PIRD

T-lymphocyte tolerance in normal homeostasis is maintained by two mechanisms; central tolerance encompasses the processes of positive and negative selection within the thymus resulting in removal of autoreactive T-lymphocytes and the development of thymic-derived regulatory T-lymphocytes (Tregs), and peripheral tolerance which includes the suppression of effector immune responses by Tregs [1, 2]. Tregs are a specialized subset of T-lymphocytes that play a pivotal role in the maintenance of self-tolerance and limiting inflammation and can be divided into two main types based on their origin: natural and inducible. Natural Tregs (nTregs) are produced within the thymus whereas inducible Tregs (iTregs) develop from naïve T-lymphocytes in the periphery upon antigen exposure. FOXP3 is a transcription factor that plays a critical role in both Treg development and suppressive function [3]. The immune regulating mechanisms of Tregs are multifarious and include suppression of effector T-lymphocyte proliferation and activation (such as by expression of CTLA4 and LRBA proteins), production of inhibitory cytokines (such as IL-10, TGFβ and IL-35) and their effect on other cell subsets such as dendritic cells and B-lymphocytes [4]. Deficiency in Treg number or function leads to a group of disorders known as ‘Tregopathies’, the prototype condition being IPEX syndrome (immune dysregulation, polyendocrinopathy, enteropathy, X-linked) caused by mutations in *FOXP3* gene. An essential component of central tolerance is the expression of Autoimmune Regulator (AIRE) transcription factor by medullary thymic epithelial cells which permits the unique ability of intra-thymic ectopic expression of a wide range of peripheral tissue-restricted self-antigens (TRAs) and to form a ‘molecular mirror of peripheral self’ [5, 6]. During the process of central tolerance, developing T-lymphocytes that react with high affinity to TRAs are deleted as they harbour the potential to elicit autoimmunity if released into the circulation. Disruption to the *AIRE* gene leads to impaired negative selection and multi-organ autoimmunity, manifesting in humans as the rare condition autoimmune polyendocrinopathy candidiasis ectodermal dystrophy (APECED) [7].

Immune dysregulation can also arise from an inability of effector immune cells to ‘switch off’ inflammation or due to alterations in cytokine signalling pathways. Hemophagocytic lymphohistiocytosis (HLH) is a life-threatening hyperinflammatory syndrome caused by impaired down-regulation of activated macrophages by natural killer (NK) cells or cytotoxic T-lymphocytes. NK cells and cytotoxic T-lymphocytes normally eliminate macrophages by formation of the immunologic synapse, insertion of a pore into the macrophage membrane, and delivery of cytotoxic granules leading to cell death. Failure to remove activated macrophages results in excessive production of inflammatory cytokines and subsequent tissue destruction. Primary HLH is caused by biallelic mutations in genes encoding proteins involved in formation of the membrane pore (Perforin, *PRF1* gene) and in cytotoxic granule formation and release mechanisms including *UNC13D*, *STX11* and *STXBP2* which encode Munc13-4, Syntaxin 11 and Munc18-2 proteins respectively. Other IEIs are associated with an increased risk of developing HLH including Chediak Higashi syndrome, Griscelli syndrome type 2 and Hermansky Pudlak syndrome [8]. Defects in immune signalling involving the JAK-STAT or NF-κB pathways alters the level of T-lymphocyte or B-lymphocyte receptor activation which impacts their development and survival and can be associated with increased autoimmunity, for example, in *STAT1* and *STAT3* gain of function mutations, and loss of function mutations in *NFKB2* presenting with an autosomal dominant combined variable immunodeficiency (CVID) phenotype. In IL-10 receptor deficiency, effector T-lymphocytes are unresponsive to the regulatory cytokine IL-10 resulting in severe early onset enteropathy [9]. Finally, inflammasomes, multiprotein complexes within the cytoplasm, constitute part of the innate immune system leading to activation of proinflammatory caspases and subsequent release of IL-1β and IL-18, and pyroptosis [10]. Dysregulated inflammasome activity is associated with monogenic autoinflammatory disorders such as familial Mediterranean fever, hyperimmunoglobulin D syndrome and chronic infantile neurological, cutaneous, and articular syndrome (CINCA).

## A diagnostic challenge

With recent huge advances in diagnostic technologies, new IEI molecular diagnoses are being discovered at an impressive rate [11]. The most recent classification from the International Union of Immunological Sciences includes 430 monogenic IEI defects with 64 new disorders described in the preceding two years with a growing proportion of these new defects falling within the PIRD category [8]. PIRD present a significant diagnostic challenge to physicians for multiple reasons. They are rare conditions; the estimated prevalence of IEIs is 1/1000–1/5000, with PIRDs representing of a subset of this. Genetic defects in the immune system are well known by physicians to be associated with serious, recurrent and/or unusual infections. However, autoimmunity, or other manifestations of immune dysregulation, either without co-existing infections or before the occurrence of infections, often does not trigger early consideration of a potential IEI, leading to a delayed or missed diagnosis. Patients may be labelled with a diagnosis such as Common Variable Immune Deficiency prior to identification of their true underlying monogenic diagnosis which may impact their management and prognosis.

There is often no correlation between genotype and phenotype and/or there is incomplete clinical penetrance in PIRD, further complicating diagnostic accuracy [12, 13]. Organ-specific autoimmunity can differ between patients with the same genetic defect. Different management strategies may be needed for individuals within the same family, for example in heterozygous loss of function mutations in cytotoxic T-lymphocyte antigen-4 (CTLA4) where family members of individuals with CTLA4-associated disease carry the same mutation and have evidence of reduced CTLA4 function but have no clinical evidence of disease, although there is potential for late onset of symptoms [12, 14, 15].

The diverse spectrum of clinical problems associated with PIRD, the frequent overlap of manifestations between different PIRD conditions and the evolution over time with gradual accrual of additional problems also contribute to the difficulty to diagnose these disorders in a timely manner. Varying degrees of activity in the residual amount of the affected protein can occur depending on the type of mutation and predisposes to significant phenotypic variability. Loss of function (LOF) or hypomorphic mutations result in a reduction or loss of protein function, whereas gain of function (GOF) or hypermorphic mutations cause enhanced protein activity. As our knowledge of monogenic IEIs expands, we can increasingly appreciate the true extent of the breadth and severity of the PIRD clinical spectrum. The classical clinical triad of IPEX syndrome (severe enteropathy, eczema and type 1 diabetes mellitus presenting early in life) is now recognized to not always be present, and patients may present with only one of these manifestations, at a later age, or with alternative autoimmune manifestations [16–18]. This diverse clinical spectrum in immune dysregulation, often predominantly involving autoimmune manifestations including cytopenias or endocrinopathies, means that patients may present to different subspecialists who may be unfamiliar with the emerging spectrum of immune dysregulatory genetic diagnoses, and patients may therefore not be offered the most appropriate treatment. This reinforces the importance of considering the ‘whole’ patient and considering an underlying IEI to be responsible for a combination of disorders afflicting the patient. Collaboration with an immunologist can help in connecting a range of medical problems to an underlying genetic defect.

First line immunological investigations including T-lymphocyte and B-lymphocyte subsets, immunoglobulin levels and vaccine responses may be normal or non-specifically abnormal in PIRD. Abnormalities in autoantibodies or inflammatory markers are also non-specific. Certain laboratory immune parameters can be suggestive of a particular disorder but not absolute in diagnosing a disease. In Autoimmune Lymphoproliferative Syndrome (ALPS) characteristic abnormalities include increased circulating CD3^+^TCRα/β^+ ^CD4^−^CD8^−^ double-negative T-lymphocytes which is highly sensitive but can also be detected in other conditions such as Systemic Lupus Erythematosus or Juvenile Idiopathic Arthritis [19–21]. Certain investigations important in the diagnosis of PIRD are not routinely available to clinicians or are performed only at a research level, which can impede the time to reach a diagnosis. Definitive diagnoses depend on genetic sequencing to identify the causative defect in PIRD.

Herein we present an overview of selected PIRDs to demonstrate the spectrum of clinical features, aspects that impede diagnosis particular to these disorders and current management strategies.

## IPEX syndrome

Hemizygous mutations in FOXP3 located on the X chromosome result in IPEX syndrome (immune dysregulation, polyendocrinopathy, enteropathy, X-linked) classically presenting in the first few weeks of life with severe enteropathy, eczema and type 1 diabetes mellitus [18]. However, prenatal presentation and late onset have been reported [17, 18, 22]. It is now recognized that patients with IPEX syndrome can present with a range of other manifestations, may be atypical with a mild phenotype, or be asymptomatic and picked up during family screening [18, 23]. Although mutations in the *FOXP3* repressor domain are associated with a more severe clinical phenotype compared to those in the leucine-zipper and forkhead domains, the same genetic defect in two different individuals can cause a different clinical presentation, suggesting the involvement of other variables that can impact the disease course and severity [23]. In a study of 96 patients with IPEX syndrome, the median time to diagnosis was 14 months after the onset of symptoms but ranged up to a maximum of almost 24 years [18]. The classical triad of enteropathy, type 1 diabetes and eczema are the most common presenting features in those with neonatal onset disease but are not always present particularly in those who present at an older age and there is a wide variety of other clinical manifestations including nephropathy, hepatitis, autoimmune hemolytic anemia, thrombocytopenia, neutropenia, thyroiditis, alopecia, food allergies and arthritis that can occur [18, 23]. First line immunological investigations may be unremarkable with the possible exception of increased eosinophils and IgE levels. T-lymphocyte and B-lymphocyte subsets are often normal. Although CD4 T-lymphocyte expression of FOXP3 may be reduced or absent, FOXP3 expression can be normal but with diminished or absent function [13]. Through international multicentre collaborative efforts, strategies to optimally manage patients with IPEX syndrome are better understood. Information from the cohort of 96 patients identified rapamycin as the preferred choice for immune suppression. In addition, this study demonstrated that although there was no difference in overall survival between those on long-term immune suppression and those who underwent allogeneic hematopoietic stem cell transplant (HSCT), transplanted patients had superior disease-free survival with stable or resolved disease, while those on immune suppression experienced disease progression [18].

## CTLA4 haploinsufficiency

CTLA4 is a negative immune regulator of T-lymphocyte proliferation and differentiation, essential in normal Treg function and maintenance of self-tolerance homeostasis [24]. CTLA4 competes with CD28 for the co-stimulatory ligands CD80 and CD86 expressed on antigen presenting cells thereby blocking co-stimulatory-dependent T-lymphocyte activation [25, 26]. Heterozygous *CTLA4* mutations cause an autosomal dominant disorder of immune dysregulation with Treg dysfunction and hyperactivation of effector T-lymphocytes [12, 14, 15] and is characterized by a highly variable clinical phenotype with different organ systems affected and incomplete clinical penetrance (approximately 70%) independent of the underlying mutation and age [15, 27]. Diagnosis is by gene sequencing followed by measuring CTLA4 protein expression and CTLA4-mediated transendocytosis. Although most affected mutation carriers present in the first two decades, late onset can also occur [15]. Initial manifestations of CTLA4 haploinsufficiency are diverse, including cytopenias, enteropathy, type 1 diabetes, skin, respiratory or neurological problems, thyroid disorders and arthritis, highlighting the likelihood of patients with this condition presenting to different specialities [15]. Autoimmunity and non-malignant lymphoproliferation are commonly found but affect different organ systems including lymph nodes, skin, lung, brain, gut, spleen and kidneys. Immunology findings are also variable but hypogammaglobulinemia is commonly observed [15, 27]. Patients with CTLA4 haploinsufficiency can benefit from targeted treatment with CTLA4 fusion proteins (abatacept or belatacept) or with mTOR inhibitors (such as sirolimus) which block CD28 signalling. Some patients undergo curative allogeneic HSCT, the most common indication being cytopenias [27, 28]. Establishing a molecular diagnosis of CTLA4 insufficiency is also important to facilitate careful monitoring of these patients who are at increased risk of malignancy [29].

## LPS-responsive beige-like anchor protein (LRBA) deficiency

LRBA deficiency is caused by biallelic mutations in *LRBA* gene, resulting in loss of or reduced expression of LRBA protein. LRBA deficiency and CTLA haploinsufficiency share a similar underlying pathophysiology; LRBA plays an essential role in recycling CTLA4 from intracellular vesicles to the cell surface and therefore impairs the availability of CTLA4 in Treg-mediated immune regulation [30]. Like other PIRDs, there is significant variability in the clinical phenotype and a lack of genotype–phenotype correlation [30–34]. Patients with residual protein expression exhibit a less severe clinical course compared to those with absent LRBA protein [34]. LRBA deficiency typically presents in the first two decades of life, although the timing of onset varies widely during this period [32]. Autoimmunity is the most common clinical manifestation, with splenomegaly, pneumonia, lymphoproliferation and enteropathy being other common clinical features [31–33, 35]. Autoimmune cytopenias are a prominent feature, but a wide spectrum of autoimmune diseases including type 1 diabetes mellitus, hepatitis, thyroid disease, and arthritis are reported and patients often exhibit polyautoimmunity [32, 33, 36]. Reduced class-switched memory B-lymphocytes, Tregs and immunoglobulin levels are the most common abnormalities in immune parameters [35]. As expected, due to the shared pathogenetic mechanisms, the phenotype of LRBA deficiency overlaps significantly with that of CTLA haploinsufficiency, although severe infections appear to be a more prominent feature in LRBA deficiency [34, 37]. A recent analysis of long-term outcomes in a large cohort of patients with LRBA deficiency demonstrated the superior effect of targeted therapy with abatacept or sirolimus with decreased disease activity scores compared to conventional immune suppression [34]. Treatment with allogeneic HSCT can result in complete disease remission, but higher disease burden pre HSCT is associated with a worse outcome [34].

## STAT3 gain of function

Signal Transducer and Activator of Transcription (STAT) 3 belongs to a transcription factor family involved in the transmission of cytokine signals including IL-6, IL-10 and IL-23 from the cell membrane to the nucleus via Janus kinases [38]. Autosomal dominant GOF *STAT3* variants cause increased transcriptional activity, impaired cytokine signalling by other STAT molecules and reduced Treg number and function [40]. Clinically it is characterized by early onset polyautoimmunity and lymphoproliferation, with immune deficiency a less prominent feature [38–40]. The clinical phenotype is diverse with incomplete penetrance and although onset is in childhood, the age at initial presentation is variable. Immunological parameters are also variable and may include T-lymphocytopenia, hypogammaglobulinemia, decreased Tregs and elevated double-negative T-lymphocytes [40]. Targeted treatment with Jakinibs (tofacitinib and ruxolitinib), small molecule inhibitors inhibiting JAK activation, has shown to be successful in STAT3 GOF disease [41]. In addition, targeted blockade of IL-6 with the anti-IL6R monoclonal antibody therapy tocilizumab has shown to be effective in STAT3 GOF [39]. Allogeneic HSCT has been used in some patients although data is limited on the effectiveness of HSCT in this specific condition [42].

## Autoimmune lymphoproliferative syndrome (ALPS)

ALPS is a disorder of immune regulation due to disruption in the process of Fas-mediated lymphocyte apoptosis. It can be caused by autosomal dominant or autosomal recessive deficiencies in several different proteins involved in this pathway; Fas (caused by germline or, less commonly, somatic mutations in *FAS* gene), FAS ligand, Caspase 8 or Caspase 10 [19, 43, 44]. There is no genotype–phenotype correlation, although later age at onset is observed in ALPS-FAS patients with a combined germline *FAS* mutation and a somatic mutation affecting the second *FAS* allele [45]. Variable clinical penetrance and differing phenotypes in patients from the same family harbouring the same mutation are observed [45, 46]. Although most cases develop symptoms in the first few years of life, adult-onset disease can also occur [45]. Patients typically present with lymphadenopathy, hepatosplenomegaly, and autoimmunity, most commonly cytopenia [45, 46]. Immunological abnormalities include elevated CD3^+^TCRα/β^+ ^CD4^−^CD8^−^ double-negative T-lymphocytes, polyclonal hypergammaglobulinemia, and increased plasma IL-10 levels [47]. Vitamin B12 is also increased. In ALPS-FAS patients, elevated Fas ligand levels are detected [47]. Whilst non-malignant lymphoproliferative disease abates with increasing age, the risk of developing autoimmune disease and lymphoma persists. Treatment is usually with high dose immunoglobulin and steroids, often with addition of a steroid-sparing agent such as sirolimus. Splenectomy is not recommended due to the lack of a sustained therapeutic response and the risk of post-splenectomy sepsis [44, 45].

## Hemophagocytic lymphohistiocytosis (HLH)

HLH is characterized by life-threatening uncontrollable hyperactivation of the immune system resulting in excessive inflammation and tissue damage, typically with multi-organ involvement. It can be triggered by a variety of factors including infections and malignancy and can occur due to underlying genetic defects as described above, resulting in failure of NK cells and cytotoxic T-lymphocytes to eliminate activated cytokine-secreting macrophages. Other IEIs have also been associated with increased incidence of HLH including Griscelli syndrome type 2, Chediak-Higashi syndrome and X-linked lymphoproliferative disease. Hemophagocytosis describes the engulfment of blood cells by activated macrophages and can be detected in tissue or bone marrow biopsies. Although finding it in tissues supports the presence of HLH, it is not pathognomic [48]. Diagnosis of HLH is challenging, particularly establishing an early diagnosis; clinical manifestations are diverse and associated with a variety of triggers, and features overlap with other potential causes such as sepsis, hepatitis, encephalitis, or Kawasaki disease [49]. Diagnosis is determined by the presence of a confirmed HLH-associated genetic mutation or the presence of at least 5 of the following 8 features: fever ≥ 38.5 °C, splenomegaly, cytopenia (in least 2 lineages), hypertriglyceridemia and/or hypofibrinogenemia, hemophagocytosis in bone marrow or tissue, low/absent NK activity, ferritin > 500 ng/mL and elevated soluble CD25 [50]. Many of these features are characteristic of an intense inflammatory response, which may not be due to an underlying genetic defect. NK cytotoxicity assays and measurement of soluble CD25, as well as other helpful immunology tests such as perforin expression and NK degranulation assays using CD107a, are not readily available in all centres and results of confirmatory genetic testing can take weeks or months in a condition where time is critical to commence treatment [49, 51]. Patients may also not meet all the defined diagnostic criteria but display clinical evidence of HLH and require HLH-targeted treatment. A high index of clinical suspicion of HLH is necessary to allow prompt diagnosis and initiation of treatment which is critical for patient survival. Conversely, the diagnosis of other inflammatory disorders or malignancy must be carefully considered and looked for to avoid inappropriate immunosuppressive treatment. First line therapy of HLH consists of etoposide and dexamethasone, with intrathecal therapy for those with CNS involvement followed by HSCT in those with an underlying predisposing genetic defect, persistent or relapsing disease [50, 52]. Alternative regimens are less toxic and utilize alemtuzumab and ciclosporin.

## Immune dysregulation with inflammatory bowel disease

Symptomatic inflammatory bowel disease (IBD) before the age of 6 years, termed ‘very early-onset IBD’ (VEOIBD), can be characterized by a severe, treatment-refractory clinical course, with predominant colonic involvement, and is more likely to have an underlying monogenic defect [53]. However, diagnosing monogenic defects causing VEOIBD is challenging due to their rarity; although the number of monogenic defects associated with VEOIBD has increased in recent years, the majority of patients with VEOIBD lack a genetic diagnosis. In addition, there is a diverse phenotypic spectrum of the IEIs associated with IBD, and currently there is a paucity of knowledge regarding many of these genetic defects. Clinical features that are more often associated with monogenic forms of IBD include young age at onset (particularly younger than two years of age), very severe disease, resistance to conventional therapies, family history of IBD or immunodeficiency, consanguinity, recurrent infections, associated autoimmunity or malignancy, or features suggestive of HLH [54]. IL-10 is an important anti-inflammatory cytokine and plays a critical role in the maintenance of immune homeostasis in the gastrointestinal tract. Loss of function mutations in IL-10 and IL10 receptor genes typically cause VEOIBD within the first few months of life, often with severe perianal involvement and is resistant to conventional immunosuppressive therapeutic approaches [55]. Allogeneic HSCT has been demonstrated to successfully cure this disease [56]. RIPK1 (receptor-interacting serine/threonine-protein kinase) is an essential signalling molecule involved in regulating cell death and inflammation. Loss of function *RIPK1* mutations are a rare cause of VEOIBD associated with a combined immunodeficiency and polyarthritis [57]. Other IEIs that are associated with colitis include IPEX syndrome, X-linked inhibitor of apoptosis protein (XIAP) deficiency, Chronic Granulomatous disease, Wiskott-Aldrich syndrome and nuclear factor κB essential modulator (NEMO) deficiency.

## Autoimmune polyendocrinopathy candidiasis ectodermal dystrophy (APECED)

Loss of functioning AIRE protein leads to defective removal of self-reactive T-lymphocytes and autosomal recessive homozygous or compound heterozygous *AIRE* mutations give rise to APECED syndrome characterized by multi-organ autoimmunity. There is no clear genotype–phenotype correlation [58] with significant clinical variability even among affected siblings The classic triad of APECED syndrome includes mucocutaneous candidiasis, hypoparathyroidism and adrenal insufficiency. However, recent studies demonstrate that the clinical spectrum of APECED is broader, encompassing more non-endocrine manifestations than previously appreciated with many patients presenting with clinical features outside the classic triad resulting in delayed diagnosis [59]. Less frequent endocrinopathies include hypothyroidism, primary ovarian or testicular failure, growth hormone deficiency and hypopituitarism. Non-endocrine manifestations include urticarial eruption, hepatitis, intestinal malabsorbtion, gastritis, tubulointerstitial nephritis, Sjogren’s-like syndrome and pneumonitis. Chronic mucocutaneous candidiasis (CMC) typically develops in the first 1–3 years of life, manifesting as recurrent, severe and chronic *Candida* infections of the skin, nails and mucous membranes but not as invasive fungal infections. A wide range of tissue-specific autoantibodies may be detected in patients with APECED and antibodies against type 1 interferons are highly sensitive and specific (> 95%) [60]. Immunological abnormalities may include increased total T-lymphocytes, CD4^+^ T-lymphocytes, CD4/CD8 ratios and reduced Treg numbers and function. Immunosuppression is needed in established autoimmune disease to prevent the development of irreversible end-organ damage, however, the evidence to guide optimal treatment strategies is limited due to the small number of patients. There is no role for HSCT in APECED syndrome as the defect originates in thymic epithelial cells and therefore will not be corrected with replacement of hematopoietic stem cells. There is no evidence supporting the routine use of prophylactic immunosuppression to prevent the development of autoimmune disease. Treatment of CMC is required to prevent complications such as oesophageal strictures, although development of resistance to oral anti-fungal agents can make long-term management of CMC challenging.

## Conclusion

Diagnosis of IEIs including PIRD is increasing largely due to continued discovery of new gene defects and improved understanding of disease presentations. Timely diagnosis of PIRD is pivotal to individualize appropriate management strategies, guide prognosis and allow genetic counselling, but is a challenge due to their rarity, diversity, and complexity. Early recognition of HLH is imperative to initiate life-saving treatment.

The diversity of presenting clinical manifestations highlights the importance of raising awareness of PIRD among different specialists, including hematology, respiratory, gastroenterology, dermatology, endocrinology, rheumatology, nephrology and neurology physicians. Specific patterns of autoimmune disorders can be associated with certain PIRD, and awareness of these patterns can help with earlier recognition or consideration of a possible monogenic cause. Clues that should alert clinicians to a potential underlying PIRD include development of autoimmunity at an age that is younger than expected, the development of multiple autoimmune processes affecting different organ systems, and/or a family history of autoimmunity or lymphoid malignancy. Early recognition and treatment of autoimmunity is essential to optimize quality of life and reduce the rate of associated complications.

Management is difficult in PIRD because of the need to balance the frequent requirement for immunosuppression and the co-existing increased risk of infection. Some PIRD such as HLH are fatal without prompt treatment. Ongoing detection of new PIRD diagnoses and worldwide collaboration and pooling of data is essential to improve our knowledge of individual rare PIRD; their underlying pathophysiology and molecular mechanisms, the clinical spectrum, natural history, and response to treatments including HSCT. Identifying those with monogenic causes allows the opportunity to use precision targeted treatment for example with the use of abatacept in CTLA4 haploinsufficiency and LRBA deficiency. As well as identifying successful therapeutic strategies, this approach also informs physicians regarding inappropriate therapeutic options for example splenectomy in the management of cytopenias which fails to result in a sustained therapeutic benefit in CTLA4 haploinsufficiency [30]. Continuing this collaborative data-sharing approach will facilitate improved individualized patient management, support development of future precision therapies and aid in identifying which patients may benefit from HSCT as well as providing important insight into the complexities underlying immune regulatory processes.

## Data Availability

Not relevant for this manuscript.

## References

[1] Hollander GA, Peterson P (2009). Learning to be tolerant: how T cells keep out of trouble. J Intern Med.

[2] Sakaguchi S (2005). Naturally arising Foxp3-expressing CD25+CD4+ regulatory T cells in immunological tolerance to self and non-self. Nat Immunol.

[3] Hori S, Nomura T, Sakaguchi S (2003). Control of regulatory T cell development by the transcription factor Foxp3. Science.

[4] Vignali DA, Collison LW, Workman CJ (2008). How regulatory T cells work. Nat Rev Immunol.

[5] Anderson MS, Venanzi ES, Chen Z, Berzins SP, Benoist C, Mathis D (2005). The cellular mechanism of Aire control of T cell tolerance. Immunity.

[6] Anderson MS, Su MA (2011). Aire and T cell development. Curr Opin Immunol.

[7] Ramsey C, Winqvist O, Puhakka L, Halonen M, Moro A, Kämpe O (2002). Aire deficient mice develop multiple features of APECED phenotype and show altered immune response. Hum Mol Genet.

[8] Tangye SG, Al-Herz W, Bousfiha A, Chatila T, Cunningham-Rundles C, Etzioni A (2020). Human inborn errors of immunity: 2019 update on the classification from the international union of immunological societies expert committee. J Clin Immunol.

[9] Kotlarz D, Beier R, Murugan D, Diestelhorst J, Jensen O, Boztug K (2012). Loss of interleukin-10 signaling and infantile inflammatory bowel disease: implications for diagnosis and therapy. Gastroenterology.

[10] Martinon F, Burns K, Tschopp J (2002). The inflammasome: a molecular platform triggering activation of inflammatory caspases and processing of proIL-beta. Mol Cell.

[11] Tangye SG, Al-Herz W, Bousfiha A, Cunningham-Rundles C, Franco JL, Holland SM (2021). The ever-increasing array of novel inborn errors of immunity: an interim update by the IUIS committee. J Clin Immunol.

[12] Schubert D, Bode C, Kenefeck R, Hou TZ, Wing JB, Kennedy A (2014). Autosomal dominant immune dysregulation syndrome in humans with CTLA4 mutations. Nat Med.

[13] d'Hennezel E, Bin Dhuban K, Torgerson T, Piccirillo CA (2012). The immunogenetics of immune dysregulation, polyendocrinopathy, enteropathy, X linked (IPEX) syndrome. J Med Genet.

[14] Kuehn HS, Ouyang W, Lo B, Deenick EK, Niemela JE, Avery DT (2014). Immune dysregulation in human subjects with heterozygous germline mutations in CTLA4. Science.

[15] Schwab C, Gabrysch A, Olbrich P, Patiño V, Warnatz K, Wolff D (2018). Phenotype, penetrance, and treatment of 133 cytotoxic T-lymphocyte antigen 4-insufficient subjects. J Allergy Clin Immunol.

[16] Hwang JL, Park SY, Ye H, Sanyoura M, Pastore AN, Carmody D (2018). FOXP3 mutations causing early-onset insulin-requiring diabetes but without other features of immune dysregulation, polyendocrinopathy, enteropathy, X-linked syndrome. Pediatr Diabetes.

[17] Zama D, Cocchi I, Masetti R, Specchia F, Alvisi P, Gambineri E (2014). Late-onset of immunodysregulation, polyendocrinopathy, enteropathy, x-linked syndrome (IPEX) with intractable diarrhea. Ital J Pediatr.

[18] Barzaghi F, Amaya Hernandez LC, Neven B, Ricci S, Kucuk ZY, Bleesing JJ (2018). Long-term follow-up of IPEX syndrome patients after different therapeutic strategies: an international multicenter retrospective study. J Allergy Clin Immunol.

[19] Sneller MC, Wang J, Dale JK, Strober W, Middelton LA, Choi Y (1997). Clincal, immunologic, and genetic features of an autoimmune lymphoproliferative syndrome associated with abnormal lymphocyte apoptosis. Blood.

[20] Mendonça LO, Matucci-Cerinic C, Terranova P, Casabona F, Bovis F, Caorsi R, et al. The challenge of early diagnosis of autoimmune lymphoproliferative syndrome in children with suspected autoinflammatory/autoimmune disorders. Rheumatology (Oxford). 2021. 10.1093/rheumatology/keab361 33909886

[21] Tarbox JA, Keppel MP, Topcagic N, Mackin C, Ben Abdallah M, Baszis KW (2014). Elevated double negative T cells in pediatric autoimmunity. J Clin Immunol.

[22] Xavier-da-Silva MM, Moreira-Filho CA, Suzuki E, Patricio F, Coutinho A, Carneiro-Sampaio M (2015). Fetal-onset IPEX: report of two families and review of literature. Clin Immunol.

[23] Park JH, Lee KH, Jeon B, Ochs HD, Lee JS, Gee HY (2020). Immune dysregulation, polyendocrinopathy, enteropathy, X-linked (IPEX) syndrome: a systematic review. Autoimmun Rev.

[24] Takahashi T, Tagami T, Yamazaki S, Uede T, Shimizu J, Sakaguchi N (2000). Immunologic self-tolerance maintained by CD25(+)CD4(+) regulatory T cells constitutively expressing cytotoxic T lymphocyte-associated antigen 4. J Exp Med.

[25] Wing K, Onishi Y, Prieto-Martin P, Yamaguchi T, Miyara M, Fehervari Z (2008). CTLA-4 control over Foxp3+ regulatory T cell function. Science.

[26] Friedline RH, Brown DS, Nguyen H, Kornfeld H, Lee J, Zhang Y (2009). CD4+ regulatory T cells require CTLA-4 for the maintenance of systemic tolerance. J Exp Med.

[27] Egg D, Rump IC, Mitsuiki N, Rojas-Restrepo J, Maccari ME, Schwab C, et al. Therapeutic options for CTLA-4 insufficiency. J Allergy Clin Immunol. 2021. 10.1016/j.jaci.2021.04.039 34111452

[28] Slatter MA, Engelhardt KR, Burroughs LM, Arkwright PD, Nademi Z, Skoda-Smith S (2016). Hematopoietic stem cell transplantation for CTLA4 deficiency. J Allergy Clin Immunol.

[29] Egg D, Schwab C, Gabrysch A, Arkwright PD, Cheesman E, Giulino-Roth L (2018). Increased risk for malignancies in 131 affected CTLA4 mutation carriers. Front Immunol.

[30] Lo B, Zhang K, Lu W, Zheng L, Zhang Q, Kanellopoulou C (2015). AUTOIMMUNE DISEASE. Patients with LRBA deficiency show CTLA4 loss and immune dysregulation responsive to abatacept therapy. Science.

[31] Azizi G, Abolhassani H, Mahdaviani SA, Chavoshzadeh Z, Eshghi P, Yazdani R (2017). Clinical, immunologic, molecular analyses and outcomes of iranian patients with LRBA deficiency: a longitudinal study. Pediatr Allergy Immunol.

[32] Gamez-Diaz L, August D, Stepensky P, Revel-Vilk S, Seidel MG, Noriko M (2016). The extended phenotype of LPS-responsive beige-like anchor protein (LRBA) deficiency. J Allergy Clin Immunol.

[33] Alkhairy OK, Abolhassani H, Rezaei N, Fang M, Andersen KK, Chavoshzadeh Z (2016). Spectrum of phenotypes associated with mutations in LRBA. J Clin Immunol.

[34] Tesch VK, Abolhassani H, Shadur B, Zobel J, Mareika Y, Sharapova S, et al. Long-term outcome of LRBA deficiency in 76 patients after various treatment modalities as evaluated by the immune deficiency and dysregulation activity (IDDA) score. J Allergy Clin Immunol. 2019. 10.1016/j.jaci.2019.12.896 31887391

[35] Habibi S, Zaki-Dizaji M, Rafiemanesh H, Lo B, Jamee M, Gámez-Díaz L (2019). Clinical, immunologic, and molecular spectrum of patients with LPS-responsive beige-like anchor protein deficiency: a systematic review. J Allergy Clin Immunol Pract.

[36] Azizi G, Abolhassani H, Zaki-Dizaji M, Habibi S, Mohammadi H, Shaghaghi M (2018). Polyautoimmunity in patients with LPS-responsive beige-like anchor (LRBA) deficiency. Immunol Invest.

[37] Gámez-Díaz L, Seidel MG (2021). Different apples, same tree: visualizing current biological and clinical insights into CTLA-4 insufficiency and LRBA and DEF6 deficiencies. Front Pediatr.

[38] Flanagan SE, Haapaniemi E, Russell MA, Caswell R, Allen HL, De Franco E (2014). Activating germline mutations in STAT3 cause early-onset multi-organ autoimmune disease. Nat Genet.

[39] Fabre A, Marchal S, Barlogis V, Mari B, Barbry P, Rohrlich PS (2019). Clinical aspects of STAT3 gain-of-function germline mutations: a systematic review. J Allergy Clin Immunol Pract.

[40] Milner JD, Vogel TP, Forbes L, Ma CA, Stray-Pedersen A, Niemela JE (2015). Early-onset lymphoproliferation and autoimmunity caused by germline STAT3 gain-of-function mutations. Blood.

[41] Forbes LR, Vogel TP, Cooper MA, Castro-Wagner J, Schussler E, Weinacht KG (2018). Jakinibs for the treatment of immune dysregulation in patients with gain-of-function signal transducer and activator of transcription 1 (STAT1) or STAT3 mutations. J Allergy Clin Immunol.

[42] Chan AY, Leiding JW, Liu X, Logan BR, Burroughs LM, Allenspach EJ (2020). Hematopoietic cell transplantation in patients with primary immune regulatory disorders (PIRD): a primary immune deficiency treatment consortium (PIDTC) survey. Front Immunol.

[43] Magerus-Chatinet A, Stolzenberg MC, Lanzarotti N, Neven B, Daussy C, Picard C (2013). Autoimmune lymphoproliferative syndrome caused by a homozygous null FAS ligand (FASLG) mutation. J Allergy Clin Immunol.

[44] Lehman HK (2015). Autoimmunity and immune dysregulation in primary immune deficiency disorders. Curr Allergy Asthma Rep.

[45] Neven B, Magerus-Chatinet A, Florkin B, Gobert D, Lambotte O, De Somer L (2011). A survey of 90 patients with autoimmune lymphoproliferative syndrome related to TNFRSF6 mutation. Blood.

[46] Price S, Shaw PA, Seitz A, Joshi G, Davis J, Niemela JE (2014). Natural history of autoimmune lymphoproliferative syndrome associated with FAS gene mutations. Blood.

[47] Oliveira JB, Bleesing JJ, Dianzani U, Fleisher TA, Jaffe ES, Lenardo MJ (2010). Revised diagnostic criteria and classification for the autoimmune lymphoproliferative syndrome (ALPS): report from the 2009 NIH International Workshop. Blood.

[48] Gars E, Purington N, Scott G, Chisholm K, Gratzinger D, Martin BA (2018). Bone marrow histomorphological criteria can accurately diagnose hemophagocytic lymphohistiocytosis. Haematologica.

[49] Jordan MB, Allen CE, Greenberg J, Henry M, Hermiston ML, Kumar A (2019). Challenges in the diagnosis of hemophagocytic lymphohistiocytosis: Recommendations from the North American Consortium for Histiocytosis (NACHO). Pediatr Blood Cancer.

[50] Henter JI, Samuelsson-Horne A, Aricò M, Egeler RM, Elinder G, Filipovich AH (2002). Treatment of hemophagocytic lymphohistiocytosis with HLH-94 immunochemotherapy and bone marrow transplantation. Blood.

[51] Rubin TS, Zhang K, Gifford C, Lane A, Choo S, Bleesing JJ (2017). Perforin and CD107a testing is superior to NK cell function testing for screening patients for genetic HLH. Blood.

[52] Henter JI, Horne A, Aricó M, Egeler RM, Filipovich AH, Imashuku S (2007). HLH-2004: diagnostic and therapeutic guidelines for hemophagocytic lymphohistiocytosis. Pediatr Blood Cancer.

[53] Kerur B, Benchimol EI, Fiedler K, Stahl M, Hyams J, Stephens M (2021). Natural history of very early onset inflammatory bowel disease in North America: a retrospective cohort study. Inflamm Bowel Dis.

[54] Uhlig HH, Schwerd T, Koletzko S, Shah N, Kammermeier J, Elkadri A (2014). The diagnostic approach to monogenic very early onset inflammatory bowel disease. Gastroenterology.

[55] Pigneur B, Escher J, Elawad M, Lima R, Buderus S, Kierkus J (2013). Phenotypic characterization of very early-onset IBD due to mutations in the IL10, IL10 receptor alpha or beta gene: a survey of the Genius Working Group. Inflamm Bowel Dis.

[56] Engelhardt KR, Shah N, Faizura-Yeop I, Kocacik Uygun DF, Frede N, Muise AM (2013). Clinical outcome in IL-10- and IL-10 receptor-deficient patients with or without hematopoietic stem cell transplantation. J Allergy Clin Immunol.

[57] Li Y, Führer M, Bahrami E, Socha P, Klaudel-Dreszler M, Bouzidi A (2019). Human RIPK1 deficiency causes combined immunodeficiency and inflammatory bowel diseases. Proc Natl Acad Sci USA.

[58] Bruserud Ø, Oftedal BE, Wolff AB, Husebye ES (2016). AIRE-mutations and autoimmune disease. Curr Opin Immunol.

[59] Ferre EM, Rose SR, Rosenzweig SD, Burbelo PD, Romito KR, Niemela JE, et al. Redefined clinical features and diagnostic criteria in autoimmune polyendocrinopathy-candidiasis-ectodermal dystrophy. JCI Insight. 2016;1(13). 10.1172/jci.insight.88782 PMC5004733 27588307

[60] Meager A, Visvalingam K, Peterson P, Möll K, Murumägi A, Krohn K (2006). Anti-interferon autoantibodies in autoimmune polyendocrinopathy syndrome type 1. PLoS Med.

